# When Blood Proteins Reveal What Transcripts Miss: Immune Cell Priming and the Road to Prognostic Biomarkers in Lupus

**DOI:** 10.1002/art.70065

**Published:** 2026-03-19

**Authors:** Ioannis Parodis

**Affiliations:** ^1^ Division of Rheumatology, Department of Medicine, Solna and Center for Molecular Medicine Karolinska Institutet and Karolinska University Hospital Stockholm Sweden; ^2^ Department of Rheumatology, Faculty of Medicine and Health Örebro University Örebro Sweden

## Introduction

Systemic lupus erythematosus (SLE) remains a disease in which we can often describe immune dysregulation with impressive molecular detail yet still struggle to predict what matters most to patients and clinicians, ie, who will accrue irreversible organ damage and when.^1^ Whole‐blood transcriptomic profiling has long provided a detailed and structured view of immune dysregulation in SLE, including modular frameworks for interpreting blood genomic data.^2^ In this issue of *Arthritis & Rheumatology*, Smith and colleagues report an integrated multi‐omic analysis in SLE that argues convincingly that serum proteomics may have the ability to illuminate clinically meaningful biology not captured by whole‐blood transcriptomics alone.^3^

## Summary of the article

Using a clinically well‐annotated National Institutes of Health (NIH) cohort of 87 patients with predominantly mild‐to‐moderate SLE and 48 healthy controls, the authors integrated high‐throughput aptamer‐based serum proteomics supported by orthogonal antibody‐based measurements, transcriptomics, immunophenotyping, and autoantibody profiling to identify patterns linked to disease activity and, importantly, long‐term damage accrual. Three protein signatures emerged from correlation structure and factor‐based scoring: (1) the interferon‐associated inflammation (IAI) signature, reflecting canonical interferon‐driven biology and correlating with established interferon gene signatures (IFNGS); (2) the granulocyte‐related (GRN) signature, capturing granulocyte activation across multiple granulocyte subsets; and (3) the immune cell priming (ICP) signature, a more unexpected constellation, enriched for molecules suggestive of enhanced costimulatory and cytokine‐mediated crosstalk between antigen‐presenting cells and effector lymphocytes, alongside markers of tissue injury, including kidney injury molecule 1^4^ and cystatin C, and immune‐related proteins such as β_2_‐microglobulin, CD40, inducible T cell 27 costimulator, interleukin 12 subunit 40, and matrix metalloproteinase 7.

Using a threshold of two SDs above the healthy control mean, almost all patients were positive for at least one protein signature, highlighting that under this operational definition, signature positivity was common in the cohort. These signatures, their key clinical associations, and the key validation steps required for translation are summarized in Table 1.

**Table 1:** Interpreting serum protein signatures in SLE: biologic framing, clinical associations, and required validation steps*

Signature	Putative biology	Key associations	Required validation steps
IAI	IFN‐associated inflammatory response	IFN biology; serologies; rash signal differences vs IFNGS	Added value over transcript‐based IFNGS; response tracking with IFN‐targeted therapy
GRN	Granulocyte activation across granulocyte subsets	Arthritis; pyuria; class IV LN; less confounding by some therapies	Relationship to NETosis/vascular injury; links to treatment response and flare prediction
ICP	ICP and tissue injury spill‐over	Kidney involvement; risk of long‐term damage accrual; limited blood gene/cell correlates	Prospective prognostic validation; paired blood and kidney tissue studies; deployable panel development

* GRN, granulocyte‐related; IAI, interferon‐associated inflammation; ICP, immune cell priming; IFN, interferon; IFNGS, interferon gene signature; LN, lupus nephritis; NETosis, neutrophil extracellular trap formation; SLE, systemic lupus erythematosus.

Two observations are particularly noteworthy. First, although multiple platforms detected associations with interferon activity and anti–double‐stranded DNA autoantibodies, whole‐blood transcriptomic alterations were not associated with organ‐specific activity measures, consistent with observations in a European SLE cohort,^5^ whereas proteins and other modalities were, most strongly with reduced glomerular filtration rate. Second, and strikingly, the ICP signature was associated with kidney involvement and with subsequent organ damage progression over approximately six years, adding prognostic information beyond conventional markers in the authors’ models. Notably, disease activity and damage indices increased with the number of protein signatures present, suggesting that cumulative “signature burden” may capture a broader immune perturbation state rather than a single pathway in isolation. The authors also report validation in an independent NIH cohort comprising 43 patients with mild‐to‐moderate SLE from 2016 to 2017, with matched controls, supporting reproducibility of signature loadings and showing consistent directionality for the prognostic association, albeit with limited power.

## Critical appraisal

A central tension in lupus biomarker research is the gap between statistical association and actionable biologic insight. The ICP signal is compelling precisely because it is not mirrored by strong circulating gene‐expression or cell‐count correlates, raising the possibility that it reflects processes occurring outside peripheral blood, with “spill‐over” into serum. The authors explore this by interrogating public kidney transcriptomic data sets, showing increased expression of several ICP‐linked genes in glomerular and tubulointerstitial compartments in patients with lupus nephritis compared with controls, and they further link a dendritic‐cell signature in kidney tissue to renal dysfunction across kidney diseases.

The mechanistic inference that ICP reflects intrarenal processes, or similar processes in other highly vascularized organs, and represents ICP within tissue is plausible but not proven. Bulk transcriptomic comparisons in archived data sets are a useful starting point, yet they cannot replace direct evidence from, for example, paired tissue and blood proteomics, spatial transcriptomics and proteomics, or functional studies.^6^, ^7^ Importantly, the study by Smith et al acknowledges the need for prospective validation and paired biopsies to establish clinical correlates and tissue relationships, which appropriately tempers overinterpretation.^3^

A second caution concerns effect size and event rates. Although the longitudinal association with damage accrual is clinically meaningful, the number of high‐damage progression events is modest, and the validation cohort is small, limitations that are common and understandable in deep multi‐omic studies but nonetheless constrain immediate clinical translation. The way forward is not to dismiss the signal but to stress test it in large and well‐characterized multicenter cohorts, ideally ethnically diverse, which would also address generalizability.

## Contextualization

The IAI signature mirrors interferon‐driven biology and correlates strongly with established IFNGS. That concordance is reassuring, and the authors note an association with rash that may differ from transcript‐based IFNGS performance in their data set.

Yet from a translational standpoint, the key question is what a protein‐based interferon signature adds beyond transcript‐based IFNGS in routine practice.^8^ The more distinctive contribution of this article may therefore be less about relabeling interferon biology and more about demonstrating a general principle: proteins can capture clinically relevant biology, as well as potentially tissue‐derived pathology, that is not readily captured by whole‐blood transcriptomics.^9^ Moreover, protein assays may be more readily translated into clinical workflows, provided that rigorous analytical validation and standardization steps are undertaken and clinically meaningful thresholds are established.

Similarly, the GRN signature usefully broadens what we mean by “neutrophil biology” in SLE by correlating with multiple granulocyte subsets and showing clinical associations with, for example, arthritis, pyuria, and class IV lupus nephritis, while appearing less confounded by certain standard therapies in this cohort. This is concordant with transcriptomic evidence of lupus nephritis–specific enrichment of neutrophil degranulation pathways when comparing active renal SLE with active nonrenal SLE in patients with no prior kidney involvement^10^ and with converging urine proteomic and blood transcriptomic data sets, as well as cross‐species transcriptomic analyses, that implicate neutrophil degranulation and neutrophil activation signatures in lupus nephritis.^11^, ^12^, ^13^ Table 1 summarizes how these signatures might be viewed as candidate biologically informed risk states, alongside the key steps needed before clinical implementation.

Beyond signatures, a major contribution of the study is the interactive SLE Immune Atlas portal, designed to allow exploration of linked clinical and multi‐omic readouts. Resources like this can accelerate the field if they (1) make cohort composition and metadata transparent, (2) support reproducible reanalysis, and (3) allow meaningful hypothesis generation across endotypes rather than only “signature look‐up.” The portal is therefore best viewed not as a clinical tool today but as a community substrate, ie, a reference data set that can be queried to help interpret future cohorts, support replication, and inform the design of prospective studies.

## Implications

To move ICP and related signatures from discovery to practice, three steps would be particularly important: first, analytical translation, which would entail moving from high‐dimensional discovery platforms to a deployable assay, eg, a small targeted protein panel, with stable calibration and prespecified thresholds; second, clinical validation, ie, demonstration of incremental prognostic value over standard‐of‐care variables, such as disease duration, baseline organ damage, proteinuria, kidney function, serologies, and treatment exposure, in larger cohorts; and third, biologic anchoring, which would connect the serum signal to tissue pathology and actionable mechanisms, especially in lupus nephritis. This would require paired sampling and spatially resolved technologies, enabling biomarkers to be related to intrarenal activity or chronicity features and response trajectories.

If successful, such an approach could shift prognostication in SLE away from single markers and toward biologically informed risk states, eg, interferon‐driven inflammation, granulocyte activation, and tissue‐linked immune priming and injury. Even if individual signatures are imperfect, their combinations may reflect the disease heterogeneity and guide enrichment strategies for clinical trials.^14^, ^15^

## Conclusions

Smith and colleagues provide a timely reminder that in SLE, where disease occurs matters as much as what we measure in blood. Their work supports the view that serum proteomics can capture a dimension of lupus biology, potentially tissue‐linked immune priming and injury, that is not faithfully represented by whole‐blood transcriptomics.

The ICP signature is best interpreted as a promising, hypothesis‐generating prognostic signal that merits careful prospective validation and deeper tissue anchoring before it can be considered clinically actionable. Done well, that next phase could bring us closer to risk‐stratified, mechanism‐informed monitoring, an outcome that people living with SLE have waited a long time to see.

## AUTHOR CONTRIBUTIONS

Dr Parodis contributed to at least one of the following manuscript preparation roles: conceptualization AND/OR methodology, software, investigation, formal analysis, data curation, visualization, and validation AND drafting or reviewing/editing the final draft. As corresponding author, Dr Parodis confirms that he has provided the final approval of the version to be published and takes responsibility for the affirmations regarding article submission (eg, not under consideration by another journal), the integrity of the data presented, and the statements regarding compliance with institutional review board/Declaration of Helsinki requirements.

## Supporting information


**Disclosure form**.
